# Optimization of the outcome of percutaneous nephrolithotomy regarding urinary leakage, what should we do?

**DOI:** 10.1007/s00240-022-01375-6

**Published:** 2022-12-02

**Authors:** Esam Abdelgawad, Ahmed M. Kadry, Khaled M. Abdelhalim, Hassan A. Abdelwahab

**Affiliations:** Urology Department, Faculty of Medicine, Suez Canal University Hospital, Round Rood, Ismailia, 41111 Egypt

**Keywords:** PCNL, Urine Leakage, Parenchymal thickness, Access tract length

## Abstract

To evaluate the factors affecting urinary leakage post percutaneous nephrolithotomy. To define those at high risk in whom a double J stent for 4 weeks or external ureteral catheter fixation for at least 3 days is indicated at the end of procedure. A total of 140 patients who underwent single-stage Percutaneous Nephrolithotomy (PCNL) with single or multiple accesses were included between February 2014 and March 2019. A detailed history, laboratory and radiological investigations were performed on all patients. All patients were classified according to postoperative urinary leakage into three groups. We defined leakage as a leakage from percutaneous puncture site. Group 1 (90 patients), No leakage was defined as leakage < 12 hours. In group 2 (32 patients), short-term leakage was defined as leakage 12-48 hours, and in Group 3 (18 patients), prolonged urinary leakage > 48 hours. Patients with short-term and prolonged urinary leakage had a significantly shorter access tract. Most patients (93.8%) with short-term leakage had an access tract of 71-90 mm, while > 50% of patients (55.6) with prolonged leakage had an access tract of 51-70 mm (*p* <0.001). Multivariate ordinal regression revealed that Operative time, length of the access tract and parenchymal thicknesses significantly predict short-term and prolonged leakage. For predicting the prolonged urinary leakage, the length of access tract and parenchymalthickness showed significant areas under the curve (AUC); 78% (95% CI: 69 – 85, *p* = 0.002) and 94% (95% CI: 87 – 97, *p* <0.001), respectively. Operative time, length of the access tract and parenchymal thickness significantly predict short-term and prolonged leakage.

## Introduction

Since first prescribed by Fernström and Johansson in 1976 Percutaneous Nephrolithotomy (PCNL) heralded a revolution in treating large renal stones [1]. It gained popularity with comparable success rates to open renal stone surgery. Now it is the recommended treatment for renal stones larger than 2 cm and staghorn stones [2]. The PCNL success rate is as high as that of open surgery. It is associated with a shorter hospital stay, less morbidity, and fewer complications than open surgery [3]. Several modifications have been made in the PCNL techniques and instruments to reduce the morbidity of the procedure and increase cost-effectiveness [4]. Urinary leakage and bleeding through the percutaneous tract are the most common complications and annoying to the surgeon before patients [5]. To overcome these problems nephrostomy catheters in different sizes are used after standard PCNL operations to secure urine drainage and to tamponade bleeding [6, 7]. In our study, we evaluated the factors that affect urinary leakage post PCNL to define those at high risk in whom double J stent or prolonged external ureteral stent is indicated.

## Materials and methods

A total of 167 consecutive patients underwent PCNL during the study period February 2014 and March 2019 at the Urology Department of Suez Canal University hospital, and data were recorded prospectively in our cross-sectional analytical study. The article was done according to the principles embodied in the Declaration of Helsinki (https://www.wma.net/what-we-do/medical-ethics/declaration-of-Helsinki/) for all investigations involving human materials and approved by the Suez Canal University ethical committee.

All patients requiring single-stage PCNL with single or multiple accesses were included in our study with a total sample of 167. Those patients who required staged PCNL, chronic renal failure (CRF), concurrent ureteric stone, UPJ obstruction, neurogenic bladder, anatomical malformations of the kidney and patients in whom double J stent was placed because of ureteropelvic injury or pelvicalyceal extravasation, obstructing residual fragments > 4 mm and tubeless PCNL were excluded from the study. After the exclusion of 27 patients, 140 patients were included in our study.

The variables of our study are considered as follows:

1. Demographic variables include history, lab, and radiological investigations.

A detailed history (age, sex, laterality, Body Mass Index (BMI), comorbidities, operative history recurrent or fresh) and laboratory investigations were performed before the procedure, including urinalysis, urine culture, a full blood count, serum creatinine, and coagulation tests. Radiological investigations include pelvic abdominal ultrasound to assess the degree of hydronephrosis and Non-Enhanced Computed Tomography (NECT) was used to measure kidney parenchymal thickness in the access line and the stones burden, number, location, and composition [Hounsfield unit (HFU) density].

The degree of hydronephrosis was defined as fissuring of the normally echogenic central renal complex (mild), dilated pelvis beyond the sinus, uniform dilated calyces, and average parenchymal thickness (moderate), and thin parenchymal thickness compared to the other side (severe) [8].

## Operative and postoperative variables by the study groups

These variables include:

Number of accesses (punctures), Length of access tract (mm), Mean HB drop (g), Need for blood transfusion, Mean operative time (min), and Postoperative outcomes: Stone free and Residual stone (non -obstructing)

The categories for “length of access tract” were selected based on Ansari et al. [1].

A prophylactic dose of 2 grams cefepime intravenous was given half an hour preoperative. In the patient’s lithotomy position, an open-ended ureteral catheter 6-F was inserted to opacify the collecting system after induction of general anesthesia.

The patient was then positioned to the prone position and a radiocontrast medium was injected to display the collecting system using fluoroscopy. Then, according to the stone distribution, subcostal puncture through the lower or middle calyx was conducted by 18 gauge puncture needles at a 30° angle, followed by tract dilatation using Alken coaxial metal dilators up to 30 F and a 30-F Amplatz sheath was inserted. A rigid 26-F nephroscope, pneumatic lithotripter, and forceps for grasping were used. With grasping forceps, fragments accessible through rigid nephroscopy were cleared. Intraoperative antegrade pyelography was used to assess collecting system integrity at the end of the procedure via a 28-F nephrostomy tube insertion into the calyx of puncture. If there were any urinary extravasation or obstructing residual fragments > 4 mm, a double J stent was inserted at the end of the procedure followed later by Extracorporeal Shock Wave Lithotripsy (ESWL).

The operative time in minutes was determined as the time from anesthesia induction to nephrostomy tube insertion. On postoperative day 1, the urethral and ureteric catheters were removed. The removal of the nephrostomy tube was determined according to the presence of hematuria and confirming the integrity of the collecting system with antegrade pyelography. When the patient complained of wet dressing, the percutaneous access site dressing was replaced. In addition, all patients’ dressings were adjusted at intervals of 6 h. Duration of Leakage (DUL) was the period between the last wet dressing and the removal of the nephrostomy tube. All patients were classified according to postoperative urinary leakage into three groups. Group 1 (90 patients), No leakage was defined as leakage < 12 hours as it did not affect the hospital stay. In group 2 (32 patients), short-term leakage was defined as leakage 12-48 hours, and in Group 3 (18 patients), prolonged urinary leakage > 48 hours.

NECT scan assessed the presence of residual stones postoperatively. A successful operation was defined as stone-free or residual stone fragment ≤ 4 mm. larger stones were known as residual stones and were measured. The mean decreases in hemoglobin and hematocrit compared to obtained values 24 hours before surgery and 36 hours after surgery, and any blood transfusions were determined.

All groups were compared regarding number and site of access puncture, length of access tract, mean hemoglobin (HB) drop, need for blood transfusion, operative time in minutes, and postoperative outcome.

In our study, we try to optimize the outcome of PCNL regarding urinary leakage by studying the most important factors affecting postoperative urinary leakage by defining those at high risk for whom double J stent for 4 weeks or external ureteral catheter fixation for at least 3 days is indicated at the end of the procedure.

### Statistical analysis

Data were entered, cleaned, and analyzed using Statistical Package for Social Sciences (IBM^®^ SPSS^®^ Statistics version 25). Categorical variables were described as frequencies and column percentages, while continuous variables were summarized as mean and standard deviation (SD). Association between the study group (i.e. Leakage) and other categorical variables were tested for significance using the Chi-square test or Fisher’s Exact test. The normality of the continuous data was tested with the Kolmogorov-Smirnov test and revealed that all continuous variables were not normally distributed. Then, the Kruskal Wallis test was used to test for the significance of the difference between the study groups.

Multivariate ordinal logistic regression model was used to identify the predictors of leakage measured on an ordinal scale from 0 to 2, where 0 was no leakage, 1 was short-term leakage, and 2 was long-term leakage. Variables entered the model include disease-related and operative variables that showed significant association or difference with study groups in bivariate analysis (e.g., parenchymal thickness, urine culture, length of access tract). Other relevant variables (e.g., stone burden, stone distribution, calyx puncture, mean operative time, and residual stones) were forced to enter the models. Hydronephrosis variables were excluded from the regression model due to a collinearity issue that violates the assumption of the regression analysis. Model fit was assessed Nagelkerke R Square, and model Chi-square test for the change in -2 Log Likelihood.

Receiver Operator Characteristics (ROC) curve was used to evaluate the performance of significant variables in the regression models (i.e., parenchymal thickness and length of access tract), for prediction of either short-term or long-term leakage. Performance of these variables was evaluated using the Area Under the Curve (AUC), Sensitivity, Specificity, positive and negative predictive values (PV). Statistical significance was identified if the *p* value was less than 0.05.

## Results

One-Hundred Forty patients were included in the analysis: 90 patients with no leakage, 32 patients with short-term leakage (<48 hours), and 18 patients with prolonged leakage ≥ 48 hours. The mean age of all patients was 45 years (**±** 11.8) and ranged from 18 to 67 years, with no statistically significant difference between the study groups. The distribution of male and female patients between the study groups showed no statistically significant difference. Most patients were overweight or obese, with no statistically significant difference between the study groups. Likewise, no statistically significant difference between the study groups regarding comorbidities and previous operative history. The mean parenchymal thickness in the short-term and prolonged leakage groups was significantly lower than the no-leakage group. Also, patients with short-term and prolonged leakage showed significantly higher percentages of moderate and marked Hydronephrosis and more positive urine cultures, compared to patients with no leakage. No statistically significant differences were detected in stone burden or number of stones, between the study groups (Table 1).

**Table 1:** Demographic and disease characteristics by study groups

Variables	No leakage (*n* = 90)	Short-term leakage (*n* = 32)	Prolonged leakage (*n* = 18)	*p* value
Age (years)	44 ± 11.1 (18–65)	46 ± 11.9 (23–66)	50 ± 13.7 (23–67)	0.086
Patient’s sex				
Male	64 (71.1%)	20 (62.5%)	12 (66.7%)	0.655
Female	26 (28.9%)	12 (37.5%)	6 (33.3%)	
BMI classification				
Normal	2 (2.2%)	2 (6.3%)	2 (11.1%)	0.171^F^
Overweight	42 (46.7%)	18 (56.3%)	6 (33.3%)	
Obese	46 (51.1%)	12 (37.5%)	10 (55.6%)	
Comorbidities (DM)	10 (11.1%)	4 (12.5%)	4 (22.2%)	0.360^F^
Positive operative history	22 (24.4%)	8 (25.0%)	4 (22.2%)	0.974
Parenchymal thickness (mm), mean ± SD	15.5 ± 2.3	12.2 ± 1.9**	10.8 ± 2.0**	< 0.001*
Hydronephrosis				
None	28 (31.1%)	0	0	< 0.001*
Mild	60 (66.7%)	2 (6.3%)	0	
Moderate	2 (2.2%)	26 (81.3%)	4 (22.2%)	
Marked	0	4 (12.5%)	14 (77.8%)	
Urine culture				
Negative	60 (66.7%)	12 (37.5%)	8 (44.4%)	0.008*
Positive	30 (33.3%)	20 (62.5%)	10 (55.6%)	
Stone burden (size in mm), mean ± SD	22.8 ± 3.6	21.4 ± 5.9	21.6 ± 2.7	0.147
Number of stones				
Single	46 (51.1%)	14 (43.8%)	10 (55.6%)	0.682
Multiple	44 (48.9%)	18 (56.3%)	8 (44.4%)	
Stone distribution				
Renal pelvis	32 (35.6%)	6 (18.8%)	4 (22.2%)	0.383
Calyx	20 (22.2%)	10 (31.3%)	6 (33.3%)	
Renal pelvis and calyx	38 (42.2%)	16 (50.0%)	8 (44.4%)	

*Statistically significant *p* value (< 0.05)

**Significant *p* value adjusted for pairwise-comparisons

^F^Fisher’s Exact test

ʺPast history of urine culture to whom antibiotics were prescribed according to culture for 10 days and procedure were postponed for at least 2 weeks until culture became negative

In Table 2, the number of punctures and calyx punctures did not show significant differences between the study groups. However, patients with short-term and prolonged leakage had a significantly shorter access tract. Many patients with short-term leakage had an access tract of 71-90 mm, while more than half of patients with prolonged leakage had an access tract of 51-70 mm. Prolonged leakage was associated with a significant drop in the mean hemoglobin level, compared to short-term leakage or no-leakage groups. However, there was no statistically significant difference in the need for blood transfusion between the study groups. Although the mean operative time was longer in patients with prolonged leakage compared to the other groups, this difference was not statistically significant. Postoperative outcomes such as stone-free rate, residual stones, fever, and bleeding were not significantly different between the study groups. However, the mean hospital stay among patients with prolonged urinary leakage was significantly longer than the other two groups.

**Table 2:** Operative and postoperative variables by the study groups

Variables	No leakage (*n* = 90)	Short-term leakage (*n* = 32)	Prolonged leakage (*n* = 18)	*p* value
Number of access (punctures)				
Single	80 (88.9%)	30 (93.8%)	14 (77.8%)	0.277^F^
Multiple (two)	10 (11.1%)	2 (6.3%)	4 (22.2%)	
Calyx puncture				
Lower	80 (88.9%)	30 (93.8%)	14 (77.8%)	0.277^F^
Lower and middle	10 (11.1%)	2 (6.3%)	4 (22.2%)	
Length of access tract (mm)				
51–70	0	2 (6.3%)	10 (55.6%)	< 0.001*^F^
71–90	56 (62.2%)	30 (93.8%)	4 (22.2%)	
> 90	34 (37.8%)	0	4 (22.2%)	
Mean ± SD	90 ± 6.6	80 ± 4.3**	78 ± 15.5**	< 0.001*^K^
Mean HB drop (g), mean ± SD	0.7 ± 1.1	0.8 ± 0.8	1.3 ± 1.3**	0.031*^K^
Need for blood transfusion	8 (8.9%)	4 (12.5%)	4 (22.2%)	0.257^F^
Mean operative time (min), mean ± SD	92.9 ± 19.6	87.8 ± 21.2	102.8 ± 25.8	0.092^K^
Postoperative outcomes				
Stone free	79 (87.8%)	26 (81.3%)	13 (72.2%)	0.220
Residual stone (non-obstructing)	11 (12.2%)	6 (18.8%)	5 (27.8.3%)	0.220
Fever	20 (22.2%)	4 (12.5%)	0	0.054
Bleeding	6 (6.7%)	2 (6.3%)	0	0.748^F^
Mean hospital stay (day)	5.7 ± 2.9	5.1 ± 1.8	8.6 ± 2.4**	< 0.001*^K^

*Statistically significant *p* value (< 0.05)

**Significant *p* value adjusted for pairwise-comparisons

^F^Fisher’s Exact test

^K^Kruskal Wallis Test

Multivariate ordinal regression revealed that Operative time, length of the access tract and parenchymal thickness significantly predict short-term and prolonged leakage, given that all other variables in the model are held constant (Table 3). Every unit-increase in the length of access tract or parenchymal thickness was significantly associated with 13.9% and 51.3%, respectively, less odds for each of short-term and long-term leakage. However, every unit-increase in the operative time was significantly associated with 3.8% higher odds for each of short-term and long-term leakage.

**Table 3:** Ordinal regression for the predictors of leakage among the studied populated

Predictors	Odds ratio	95% CI	*p* value
Stone burden (size in mm)	0.995	0.900–1.101	0.927
Stone distribution			
Renal pelvis and calyx	1	0.736–8.156	0.144
Calyx	2.450	0.321–3.624	0.903
Renal pelvis	1.078		
Positive urine culture	2.00	0.666–6.024	0.216
Calyx puncture			
Middle/multiple vs. lower	0.160	0.027–1.035	0.052
Operative time (min)	1.038	1.010–1.066	0.007*
Length of access tract (mm)	0.861	0.807–0.919	< 0.001*
Parenchymal thickness (mm)	0.487	0.367–0.647	< 0.001*
Residual stone	0.527	0.151–1.842	0.316

Nagelkerke *R*^2^ = 0.67; Model Fit Chi-square = 112.85, df = 9, *p* value < 0.001)

*Statistically significant *p* value (< 0.05)

**Fig. 1:**
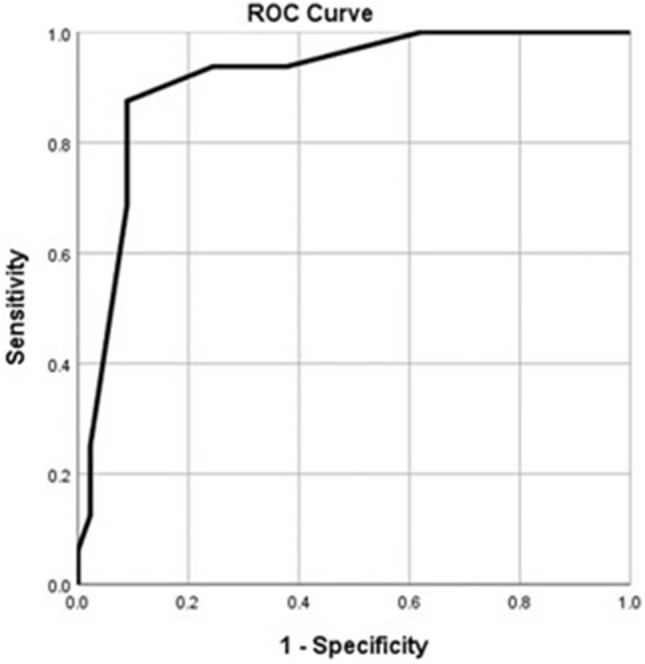
ROC curve of the length of access tract (mm) for predicting short term leakage. AUC = 0.91 (95% CI: 0.85 – 0.96; *p*<0.001)

**Fig. 2:**
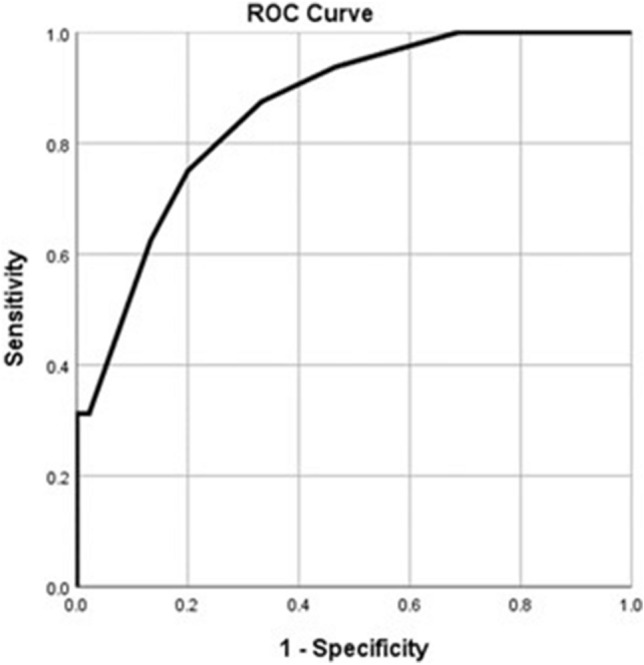
ROC curve of the parenchymal thickness for predicting short-term leakage. AUC = 0.86 (95% CI: 0.79 – 0.93; *p*<0.001)

ROC curves were performed for the significant predictors (i.e., the length of access tract and parenchymal thickness). For predicting the short-term urinary leakage, the length of access tract and parenchymal thickness showed significant areas under the curve (AUC); 91% (95% CI 85–96, *p* < 0.001) and 86% (95% CI 79–93, *p* < 0.001), respectively (Figs. 1 and 2). The best cut-off value of the length of the access tract is less than or equal to 84 mm with 87.5% sensitivity, 91.1% specificity, 77.8% positive predictive value, and 95.3% negative predictive value. The best cut-off value of the parenchymal thickness is less than or equal to 13 mm with 75% sensitivity, 80% specificity, 57.1% positive predictive value, and 90% negative predictive value.

**Fig. 3:**
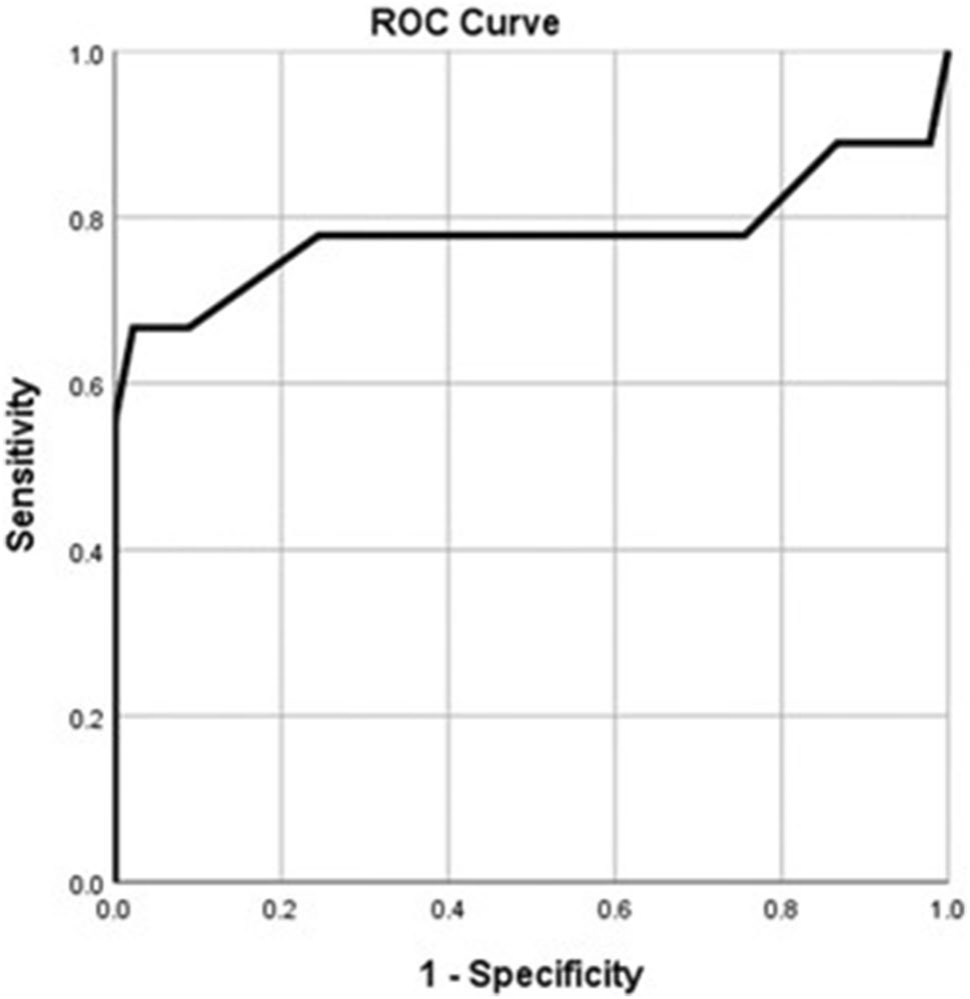
ROC curve of the length of access tract (mm) for predicting long-term leakage. AUC = 0.78 (95% CI: 0.69 – 0.85; *p*=0.002)

**Fig. 4:**
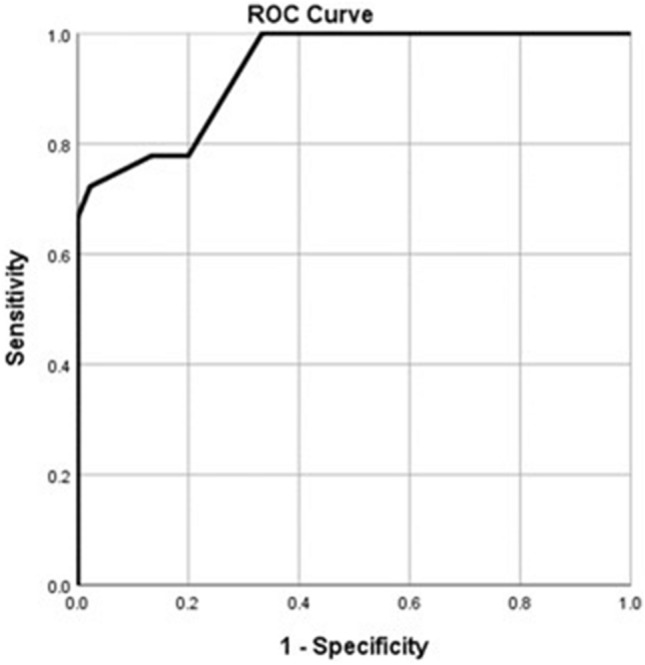
ROC curve of the parenchymal thickness (mm) for predicting long-term

For predicting the prolonged urinary leakage, the length of access tract and parenchymal thickness showed significant areas under the curve (AUC); 78% (95% CI 69–85, *p* = 0.002) and 94% (95% CI 87–97, *p* < 0.001), respectively (Figs. 3 and 4). The best cut-off value of the length of the access tract is less than or equal to 75 mm with 66.7% sensitivity, 97.8% specificity, 85.7% positive predictive value, and 93.6% negative predictive value. The best cut-off value of the parenchymal thickness is less than or equal to 11 mm with 72% sensitivity, 97.8% specificity, 86.7% positive predictive value, and 94.6% negative predictive value.

## Discussion

PCNL is the treatment modality of choice for most renal stones larger than 2 cm and complex renal stones [9]. Modified Clavien grading system is a reliable classification system for comparison of post PCNL complication, Tefekli et al. [3]. used the modified Clavien grading system, urinary leakage from the nephrostomy site for < 12 h was considered as Grade II complication while the double J stent placement for urine leakage more than 48 h was considered as Grade III complication. Our study also showed that prolonged urinary leakage that requires a double J stent, is the most common type of Grade IIIA complication.

Percentage of urinary leakage was (35%) in our study while Dirim et al. [10]. reported urinary leakage in 70.2% of patients which was higher than that of our results because they defined leakage if it was more than 6 hours, not 12 hours as it was in our study.

The need for a double J stent because of prolonged urinary leakage more than 48 hours Post PCNL was 13% in our study while Binbay et al. [11]. reported that between 4.3% and 5% of patients required double J stent which was lower than that of our study, because they did not report about the parenchymal thickness in their study which was the most important risk factor for Prolonged urinary leakage in our study while our results were comparable with that of Dirim et al. [10] which was 14%, while Ansari et al. [1] reported that 5.5% of patients underwent double J stent placement.

In our study, we found that Neither gender, age, comorbidities, stone number nor stone distribution, had a significant effect on urine leak post PCNL. Also, Dirim et al. [10] reported the same results.

Our study concluded that BMI did not affect the presence and duration of urinary leakage after PCNL which was consistent with other studies that also reported that BMI had no impact on the complication rates of PCNL [10, 12, 13]. Faerber and Goh have noted a higher complication rate and longer hospital stay in obese cases when compared to normal-weight patients undergoing PCNL [14].

We found that stone burden did not influence post-PCNL prolonged urinary leakage, Uyeturk et al. [2], Ansari et al. [1], and Dirim et al. [10] demonstrated the same results, while Binbay et al. [11] reported that stone burden prolongs the duration of hospital stay due to prolonged urinary leakage**.**

In our study, it was found that positive surgical history did not affect post-PCNL urinary leakage. Also, Margel et al. [15] Ansari et al. [1] , Uyeturk et al. [2], and Dirim et al. [10] demonstrated that PCNL can be performed successfully without increased risk of complications in patients with a history of positive surgical history or ESWL.

In the present study, as well as Dirim et al. [10], Binbay et al. [11], Uyeturk et al. [2], and Ansari et al. [1] there was a positive correlation between the degree of renal hydronephrosis and prolonged urinary leakage and the duration increased with the increase in the degree of hydronephrosis.

It has been reported that reduced parenchymal thickness favors less blood loss. However, the impact on urine leakage has not been addressed [16]. It can only be speculated that thinner parenchyma has lost its compressive and thus the sealing effect which in turn causes increased urine leak.

The present study confirmed that renal parenchymal thickness in the access line was inversely correlated with Post PCNL urinary leakage. Uyeturk et al. [2] and Ansari et al. [1] reported that renal parenchymal thickness in access lines is highly correlated with the duration of urinary leakage.

In the present study, no relationship was found between the postoperative drop in Hb level and prolonged urinary leakage but there was a statistically significant difference between the different groups in our study regarding Postoperative HB drop.

Fever secondary to UTI is one of the most common complications of PCNL [17]. In our study, fever was not found to be influencing the prolonged urinary leakage.

Our study as well as that of Dirim et al. [10] found no relation between the outcome of surgery in form of stone-free rate, residual stones, and the duration of Post PCNL urinary leakage, while Binbay et al. demonstrated that post PCNL residual stone fragments increased the duration of urinary leakage [11]. Our patients with clinically significant residual stones were managed by ESWL while we excluded patients with larger stones in need of a second look.

We found that the operative time was a positive predictor of post-operative prolonged urinary leakage, in contrast to Uyeturk et al. [2], Ansari et al. [1], Dirim et al. [10] and Binbay et al. [11] who demonstrated that the operative time is not a predictor of prolonged urinary leakage. Our results could be supported by the explanation that longer operations could influence the inflammatory indices and enhance the risk of pelvicalyceal edema [18], Moreover, long time exposure to irrigation fluids may affect the risk of leakage [11].

We found that tract length negatively affects the post PCNL urinary leakage while Ansari et al. [1], Uyeturk et al. [2] reported that the Distance from the skin to desired calyx puncture (tract length) does not affect prolonged urinary leakage, this may be because Ansari et al, did not assess parenchymal thickness in their study.

Limitation of our study were the small sample size, and the exclusion of patients with impaired kidney function and concurrent ureteric stones. We excluded patients with prefixed DJ intraoperative as it will be a confounding factor and it could affect postoperative urinary leakage.

## Conclusion

Operative time, length of the access tract and parenchymal thickness significantly predict short-term and prolonged leakage. For predicting prolonged urinary leakage from a percutaneous nephrostomy site following a PCNL, consider a cut-off value of the length of the access tract is ≤ 75 mm (and/or a cut-off value of the parenchymal thickness is ≤ 11 mm).

## Data Availability

All data are available upon request and approval from the department of medical records at Suez Canal University Hospital.
